# Prenatal hypertension as the risk of eclampsia, HELLP syndrome, and critical obstetric hemorrhage

**DOI:** 10.1038/s41440-023-01511-8

**Published:** 2023-11-22

**Authors:** Tetsuya Akaishi, Kunio Tarasawa, Hirotaka Hamada, Noriyuki Iwama, Hasumi Tomita, Miho Akaishi, Kiyohide Fushimi, Kenji Fujimori, Nobuo Yaegashi, Masatoshi Saito

**Affiliations:** 1 Department of Education and Support for Regional Medicine, Tohoku University Hospital, Sendai, Japan; 2 Department of Health Administration and Policy, Tohoku University Graduate School of Medicine, Sendai, Japan; 3 Department of Obstetrics and Gynecology, Tohoku University Graduate School of Medicine, Sendai, Japan; 4 Department of Health Policy and Informatics, Tokyo Medical and Dental University Graduate School of Medical and Dental Sciences, Tokyo, Japan; 5 Tohoku Medical Megabank Organization, Tohoku University, Sendai, Japan

**Keywords:** Critical obstetrical hemorrhage, HELLP syndrome, Prenatal hypertension, Maternal mortality rate, Red blood cell transfusion

## Abstract

Critical bleeding is a common cause of maternal mortality in obstetric patients. However, the non-obstetric factors underlying critical obstetric bleeding remain uncertain. Therefore, this study aimed to clarify the impact of chronic hypertension on obstetric hemorrhage by evaluating a nationwide administrative database in Japan. Women who gave birth between 2018 and 2022 were enrolled. The primary outcome was critical hemorrhage requiring massive red blood cell (RBC) transfusion during childbirth. In total, 354, 299 eligible women were selected from the database. The maternal mortality rate was >1.0% among those who received a massive RBC transfusion (≥4000 cc), and this amount was used as the cutoff of the outcome. Critical hemorrhage was less frequent with elective Caesarean section (CS) compared with vaginal childbirth or emergent CS (odds ratio [OR], 0.38; 95% confidence interval, 0.30–0.47). Multiple logistic regression analysis adjusting for these obstetric risks revealed that a higher maternal age (adjusted OR [aOR] per 1 year, 1.07 [1.05–1.09]); oral medications with prednisolone (aOR, 2.5 [1.4–4.4]), anti-coagulants (aOR, 10 [5.4–19]), and anti-platelets (aOR, 2.9 [1.3–6.4]); and a prenatal history of hypertension (aOR, 2.5 [1.5–4.4]) and hypoproteinemia (aOR, 5.8 [1.7–20]) are the risks underlying critical obstetric hemorrhage. Prenatal history of hypertension was significantly associated with obstetric disseminated intravascular coagulation (OR, 1.9 [1.5–2.4]); Hemolysis, Elevated Liver enzymes, and Low platelet count (HELLP) syndrome (OR, 3.3 [2.7–4.2]); and eclampsia (OR, 6.1 [4.6–8.1]). In conclusion, a maternal prenatal history of hypertension is associated with the development of HELLP syndrome, eclampsia, and resultant critical hemorrhage.

**Figure:**
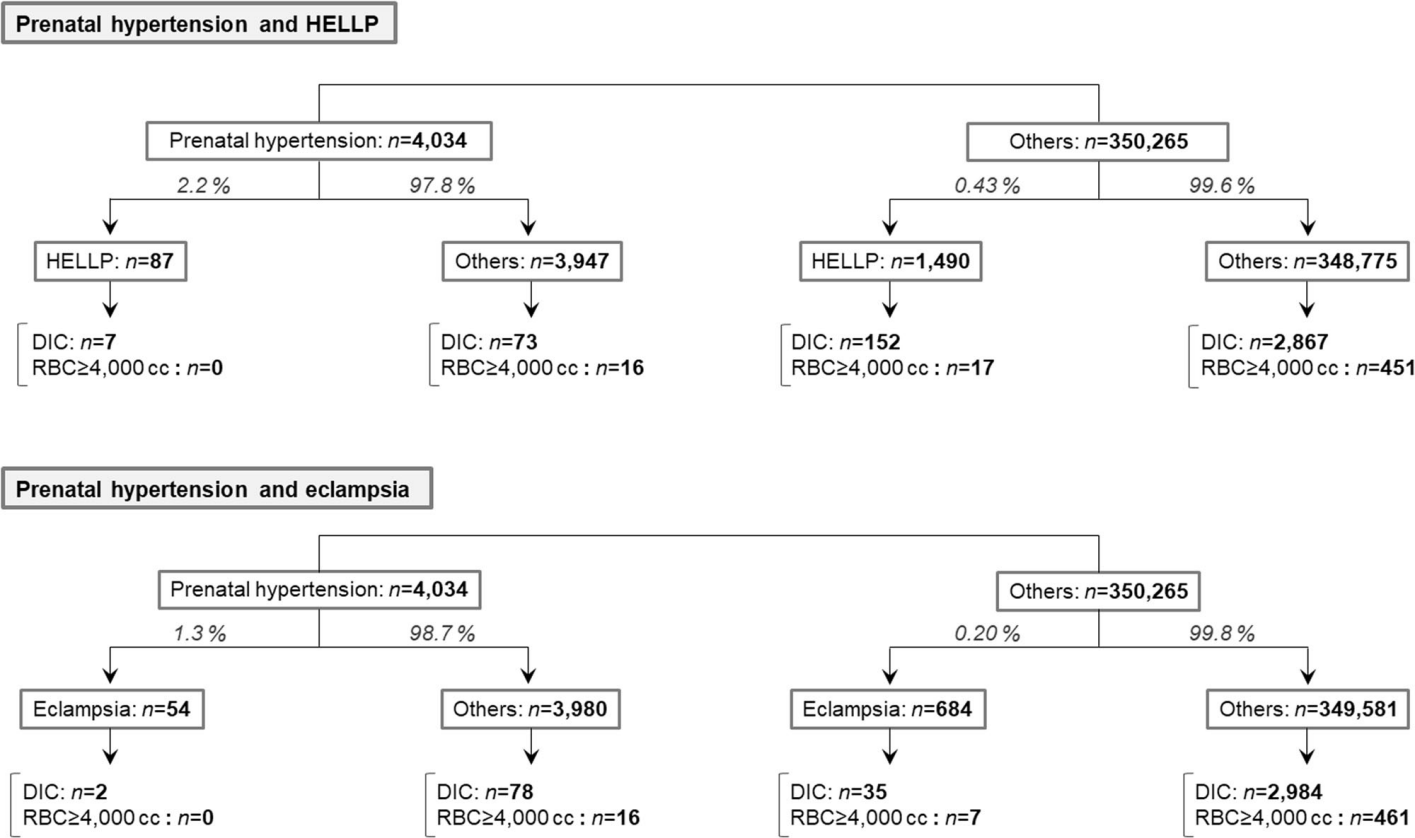
The incidence of HELLP syndrome and eclampsia increased more than fivefold in the presence of prenatal hypertension. However, the likelihood of subsequently developing DIC or experiencing critical bleeding did not change by the presence of prenatal hypertension.

The incidence of HELLP syndrome and eclampsia increased more than fivefold in the presence of prenatal hypertension. However, the likelihood of subsequently developing DIC or experiencing critical bleeding did not change by the presence of prenatal hypertension.

## Introduction

Critical bleeding in obstetrics is a major cause of maternal death during pregnancy worldwide, accounting for approximately 300,000 deaths globally [1]. With advances in obstetric critical care and treatment over the last several decades, maternal mortality has significantly decreased [2]. However, some women still lose their lives during childbirth because of miscellaneous complications like critical bleeding, intracranial hemorrhage, and sepsis. Among these, maternal death due to critical bleeding decreased by approximately 50% between 2010 and 2020 in the country [3]. This reduction could be partly attributed to the “Guidelines for management of critical bleeding in obstetrics,” published by Japan Society of Obstetrics and Gynecology and other four related academic societies in 2010 [4–6]. This guideline emphasized the difficulty in accurately measuring the amount of bleeding during childbirth and incorporated an estimator of blood loss based on vital signs, namely, the Shock Index (SI) calculated as the heart rate divided by the systemic blood pressure [7, 8]. An SI > 1.5 implies the presence of critical blood loss of >2500 cc [5], which is a life-threatening condition and is considered as critical bleeding in obstetrics [9–11]. Although the possibility of overtriage exists in implementing this criterion, the guidelines could have contributed to reducing maternal death in the last decade in Japan. Many obstetric conditions are known risk factors for critical bleeding, such as amniotic embolism, uterine rupture, placental abruption, atonic bleeding, placenta accreta, and uterine inversion [12–15]. However, the prenatal non-obstetric risks of critical maternal bleeding have not been widely investigated. Conceivable non-obstetric prenatal risks underlying critical bleeding may include chronic hypertension, diabetes mellitus, and other conditions requiring the use of steroids, anticoagulants, and antiplatelets. As the median maternal age at childbirth has gradually increased in many developed countries [16, 17], the importance of controlling non-obstetric risks underlying critical maternal bleeding has increased [18]. Therefore, the present study aimed to identify the prenatal non-obstetric risks contributing to the development of critical obstetric bleeding, especially focusing on prenatal history of hypertension, by retrospectively investigating data from a nationwide administrative database covering the majority of tertiary care hospitals in the country.

## Methods

### Data source and participants

This study used the Japanese Diagnosis Procedure Combination (DPC)-based payment system, a nationwide administrative database. The evaluated population in this study comprised women who delivered their children between April 2018 and March 2022 at more than 1100 hospitals that joined the DPC payment system and agreed to cooperate with the present surveillance. Only childbirths with a certain delivery date were evaluated as the primary objective was to evaluate the risk of critical bleeding requiring RBC (red blood cell) transfusion on the same day of childbirth.

### Evaluated variables

Data on the use and the amount of RBC transfusions on the day of childbirth were collected from the enrolled women. Using death as the outcome and stratifying the maternal mortality rate according to the amount of RBC transfusion, we attempted to determine the optimal cut-off for defining critical bleeding in obstetrics based on the administered amount of RBC transfusion. The evaluated demographic data included maternal age and body mass index (BMI) at childbirth. The evaluated obstetric conditions included the type of child delivery (vaginal, elective Caesarean section [CS], and emergent CS), total hysterectomy, and presence of the following complications during the childbirth process: hypertensive disorders of pregnancy (HDP) regardless of prenatal history of hypertension [19, 20]; disseminated intravascular coagulation (DIC); Hemolysis, Elevated Liver enzymes, and Low platelet count (HELLP) syndrome [21]; placental abruption; amniotic fluid embolism; low-lying placenta (LLP) and placenta previa (PP); placenta accreta; uterine rupture; eclampsia; atonic bleeding; uterine inversion; and birth canal laceration. The evaluated prenatal non-obstetric conditions included a history of hypertension, diabetes mellitus, iron deficiency anemia, hypoproteinemia/hypoalbuminemia, and prenatal use of the following medications: oral prednisolone, oral anticoagulants, or oral antiplatelets. These prenatal non-obstetric conditions were confirmed to have occurred and were registered in the DPC database before hospitalization for childbirth. Therefore, none of the prenatal non-obstetric conditions evaluated in this study occurred during or after critical hemorrhage.

### Statistical analysis

The distributions of age and BMI are described as medians and interquartile ranges (IQR; 25–75 percentiles). Comparisons of quantitative data between the two groups were performed using the Mann–Whitney *U* test. Comparisons of the frequencies between the two groups were performed using the chi-square test or Fisher’s exact test, according to the number of individuals in each subgroup. Effect size was reported as an unadjusted odds ratio (OR) together with the 95% confidence interval (CI). OR of variables with complete separation was calculated by a Firth’s logistic regression analysis based on a penalized maximum likelihood estimation using R package “logistf [22].” To calculate the adjusted OR (aOR) in multiple binary logistic regression analyses, the occurrence of critical obstetric bleeding, which was defined as an administration of an RBC transfusion with the amounts greater than the cutoff level determined from maternal mortality rate, was considered as the outcome. Multiple binary logistic regression analyses were performed on the overall cohort (354,299 women) and 79,731 women who had vaginal childbirth. In the former regression model for the overall population, the type of delivery was used as an explanatory variable and incorporated into the model using dummy variables. In each logistic regression model, variables with two-sided *p*-values < 0.05 in the univariate analyses or those with particular clinical interests (i.e., age, BMI, and non-obstetric prenatal factors) were selected as the explanatory variables. Statistical analyses were performed using the R Statistical Software version 4.1.3 (R Foundation for Statistical Computing, Vienna, Austria).

## Results

### Population characteristics

From the DPC database, which incorporates 30,557,174 cumulative patients who were hospitalized between April 2018 and March 2022, we enrolled 354,299 cumulative women with a known date of childbirth during the study period in the present study. The median age, height, weight, and BMI at childbirth was 33 (IQR: 30–37) years, 158 (154–162) cm, 61.7 (55.8–68.9) kg, and 24.7 (22.5–27.5), respectively. Among the cumulative 354,299 women who delivered, 10,119 (2.9%) required an RBC transfusion on the day of the childbirth. Among the 10,119 women who required an RBC transfusion, 5890 (58.2%) were simultaneously treated with fresh frozen plasma transfusion. All-cause maternal mortality rate was 0.011% (*n* = 38/354,299) among the evaluated overall cohort and 0.28% (*n* = 28/10,119) among those who required an RBC transfusion.

### Cutoff level of RBC transfusion amount

Among the 10,119 women who received an RBC transfusion, the frequency of all-cause maternal death, stratified by the amount of the RBC transfusion administered, is shown in Table 1. The estimated maternal mortality rate was >1.0% in women who required an RBC transfusion of ≥4000 cc. This rate in women who required an RBC transfusion of ≥4000 cc was approximately 1000 times higher than that among those who did not require an RBC transfusion. The estimated mortality rates were more than 10 times higher in those who required an RBC transfusion of ≥4000 cc compared with those who required an RBC transfusion of <4000 cc. Based on these findings, peripartum events requiring an RBC transfusion of ≥4000 cc (*n* = 484) were regarded as life-threatening critical bleeding events in obstetrics. With this cutoff level, women who required a massive RBC transfusion comprised 4.8% of those who required an RBC transfusion and 0.14% of the overall population. For reference, the majority of women who were administered an RBC transfusion of 10,000–19,800 cc (93.5%) survived, whereas only 20% of those who required an RBC transfusion of ≥20,000 cc survived (*p* = 0.0012, Fisher’s exact test).

**Table 1:** All-cause maternal mortality rate stratified by the amount of red blood cell transfusion

Amount of RBC transfusion [mL]	Total women, *n*	Maternal death, *n* (%)	
		*n*	% (95% CI)
None	344,180	10	0.0029 (0.0016–0.0053)%
200–800 cc	5621	6	0.11 (0.049–0.23)%
1000–1800 cc	2568	2	0.078 (0.021–0.28)%
2000–2800 cc	1130	4	0.35 (0.14–0.91)%
3000–3800 cc	316	2	0.63 (0.17–2.3)%
4000–4800 cc	215	3	1.4 (0.48–4.0)%
5000–5800 cc	92	2	2.2 (0.60–7.6)%
6000–6800 cc	78	1	1.3 (0.23–6.9)%
7000–7800 cc	33	1	3.0 (0.54–15.3)%
8000–8800 cc	22	0	0.0 (0.0–14.9)%
9000–9800 cc	8	1	12.5 (2.2–47.1)%
10,000–19,800 cc	31	2	6.5 (1.8–20.7)%
≥20,000 cc	5	4	80.0 (37.6–96.4)%
200–3800 cc	9635	14	0.15 (0.087–0.24)%
≥4000 cc	484	14	2.9 (1.7–4.8)%

The estimated maternal mortality rate increases to >1.0% in women with critical bleeding that required an RBC transfusion of ≥4000 cc. This rate was more than 100 times higher than the maternal mortality rate among women who did not require RBC transfusions. The estimated mortality rates were more than 10 times higher in those who required an RBC transfusion of ≥4000 cc compared with those who required an RBC transfusion of <4000 cc

*CI* confidence interval, *RBC* red blood cell

### Impact of age and BMI on the occurrence of critical bleeding

The median (IQR) maternal age was significantly higher in the 484 women who required an RBC transfusion of ≥4000 cc compared with the other 353,815 women (35.5 [32–39] years *vs* 33 [30–37] years; *p* < 0.0001 with Man–Whitney *U* test; effect size *r* = 0.01). BMI was significantly lower in women with critical bleeding who required an RBC transfusion of ≥4000 cc than in the rest of the cohort (median [IQR]:24.2 [21.7–26.8] vs 24.7 [22.5–27.5]; *p* < 0.0001; effect size *r* = 0.01).

### Overview of the conditions underlying critical bleeding

First, the prevalence of critical bleeding that required an RBC transfusion of ≥4000 cc between those with and without the following variables was compared using univariate analyses: platelet transfusion, FFP transfusion, DIC, HELLP syndrome, maternal death, total hysterectomy, and each of the three types of childbirth (i.e., vaginal childbirth, elective CS, and emergent CS) (Table 2). The unadjusted ORs differed between those who had vaginal childbirth (OR, 1.75; 95% CI, 1.44–2.12), an elective CS (OR, 0.38; 95% CI, 0.30–0.47) and an emergent CS (OR, 1.50; 95% CI, 1.25–1.80). Therefore, we decided to perform subsequent analyses to identify the risk of critical bleeding after stratification by delivery type. The risk of critical bleeding was further compared among the three types of childbirth using multiple binary logistic regression analysis, with age, BMI, and type of delivery as the explanatory variables. As a result, elective CS showed aORs <1.0 when compared with vaginal delivery (aOR, 0.32; 95% CI, 0.25–0.41) or emergent CS (aOR, 0.38; 95% CI, 0.30–0.48). The odds of critical bleeding did not significantly differ between vaginal delivery and emergent CS (OR, 1.18; 95% CI, 0.95–1.45). Regarding the non-obstetric conditions, prenatal history of hypertension (OR, 2.98; 95% CI, 1.69–4.89) and hypoproteinemia (OR, 23.8; 95% CI, 6.35–62.8) and the use of oral prednisolone (OR, 3.59; 95% CI, 2.07–5.82), anti-coagulants (OR, 11.6; 95% CI, 6.09–20.1), or anti-platelets (OR, 5.66; 95% CI, 2.42–11.3) had significant ORs (>1.0) for the occurrence of critical bleeding.

**Table 2:** Obstetric and non-obstetric conditions underlying critical bleeding requiring an RBC transfusion of ≥ 4000 cc

Characteristics	RBC transfusion ≥ 4000 cc				OR (95% CI)^a^	*P*-value
	Yes (*n* = 484)		No (*n* = 353,815)			
	With (*n*)	Without (*n*)	With (*n*)	Without (*n*)		
Maternal death	14	470	24	353,791	438 (209–904)	<0.0001
Total hysterectomy	73	411	107	353,708	584 (428–830)	<0.0001
HDP	13	471	4708	349,107	2.05 (1.08–3.54)	0.0164
DIC	313	171	2786	351,029	230 (189–281)	<0.0001
HELLP	17	467	1560	352,255	8.22 (4.74–13.3)	<0.0001
Vaginal childbirth	163	321	79,568	274,247	1.75 (1.44–2.12)	<0.0001
Elective CS	106	378	151,297	202,518	0.38 (0.30–0.47)	<0.0001
Emergent CS	215	269	122,950	230,865	1.50 (1.25–1.80)	<0.0001
Vacuum extraction	52	432	47,386	306,429	1.03 (0.76–1.38)	0.8864
Episiotomy	14	470	12,411	341,404	0.82 (0.44–1.39)	0.5408
Oral prednisolone	17	467	3149	350,666	4.05 (2.34–6.57)	<0.0001
Oral anti-coagulants	13	471	842	352,973	11.6 (6.09–20.1)	<0.0001
Oral anti-platelets	8	476	1039	352,776	5.71 (2.44–11.4)	0.0001
Oral EPA	0	484	42	353,773	8.59 (0.07–60.2)^b^	0.2613
Prenatal hypertension	16	468	4018	349,797	2.98 (1.69–4.89)	<0.0001
Diabetes mellitus	4	480	4485	349,330	0.65 (0.18–1.67)	0.5394
IDA	75	409	56,246	297,569	0.97 (0.75–1.24)	0.8521
Hypoproteinemia	4	480	124	353,691	23.8 (6.35–62.8)	0.0003

*CS* Caesarean section, *DIC* disseminated intravascular coagulation, *HDP* hypertensive disorders of pregnancy, *HELLP* hemolysis, elevated liver enzymes, and low platelet count, *IDA* iron deficiency anemia, *OR* odds ratio, *RBC* red blood cell

^a^Unadjusted OR

^b^Firth’s logistic regression analysis was performed for these variables with complete separation

### Obstetric complications and critical bleeding with respect to the type of delivery

Based on the aforementioned finding of different occurrence rates of critical bleeding associated with the three types of childbirth, univariate analyses with obstetric complications for the occurrence of critical bleeding were performed after dividing the population based on delivery type (Table 3). ORs calculated using Firth’s logistic regression analyses of variables with complete separation are shown only for reference. Among the 79,731 women who had vaginal childbirth, HELLP syndrome, amniotic embolism, LLP/PP, placenta accreta, uterine rupture, atonic bleeding, and uterine inversion had significant ORs (>1.0) for the development of critical bleeding. Among the 151,403 women who had an elective CS, HELLP syndrome, amniotic embolism, LLP/PP, placenta accreta, and atonic bleeding had significant ORs (>1.0). Among the 123,165 women who had an emergent CS, HDP, HELLP syndrome, placental abruption, LLP/PP, placenta accreta, uterine rupture, eclampsia, and atonic bleeding had significant ORs (>1.0).

**Table 3:** Univariate analyses with obstetric complications for the occurrence of critical bleeding by the type of delivery

Characteristics	Critical bleeding, *n* (%)	OR (95% CI)^a^	*P*-value
Vaginal childbirth (*n* = 79,731, including 163 [0.20 %] cases with critical bleeding)			
HDP	1/914 (0.11%)	0.532 (0.013–3.01)	>0.99
HELLP	2/72 (2.78 %)	14.1 (1.66–53.8)	0.0097
Placental abruption	2/375 (0.53 %)	2.64 (0.316–9.75)	0.1790
Amniotic embolism	14/30 (46.67 %)	460 (207–1090)	<0.0001
LLP and PP	4/261 (1.53 %)	7.76 (2.07–20.5)	0.0021
Placenta accreta	33/2126 (1.55 %)	9.40 (6.19–13.9)	<0.0001
Uterine rupture	8/19 (42.11 %)	370 (129–1034)	<0.0001
Eclampsia	1/203 (0.49 %)	2.43 (0.061–13.9)	0.3400
Atonic bleeding	112/14,630 (0.77 %)	9.84 (7.00–14.0)	<0.0001
Uterine inversion	9/109 (8.26 %)	46.4 (20.3–93.9)	<0.0001
Birth canal laceration	20/8798 (0.23 %)	1.13 (0.668–1.81)	0.7049
Elective CS (*n* = 151,403, including 106 [0.07 %] cases with critical bleeding)			
HDP	2/1378 (0.15%)	2.10 (0.250–7.78)	0.2512
HELLP	1/50 (2.00 %)	29.4 (4.02–215)	0.0344
Placental abruption	0/47 (0.00 %)	15.0 (0.118–105)^b^	0.1840
Amniotic embolism	4/19 (21.1 %)	395 (93.7–1302)	<0.0001
LLP and PP	52/10,956 (0.47 %)	12.4 (8.30–18.5)	<0.0001
Placenta accreta	10/637 (1.57 %)	25.0 (11.6–48.4)	<0.0001
Uterine rupture	0/29 (0.00 %)	24.1 (0.190–173)^b^	0.1369
Eclampsia	0/20 (0.00 %)	34.6 (0.272–254)^b^	0.1096
Atonic bleeding	65/14,084 (0.46 %)	15.5 (10.3–23.6)	<0.0001
Uterine inversion	0/12 (0.00 %)	56.8 (0.442–436)^b^	0.0814
Birth canal laceration	0/3 (0.00 %)	203 (1.51–2111)^b^	0.0393
Emergent CS (*n* = 123,165, including 215 [0.17 %] cases with critical bleeding)			
HDP	10/2419 (0.41%)	2.43 (1.16–4.57)	0.0109
HELLP	14/1455 (0.96 %)	5.87 (3.15–10.1)	<0.0001
Placental abruption	17/3694 (0.46 %)	2.78 (1.59–4.58)	<0.0001
Amniotic embolism	13/30 (43.3 %)	469 (205–1020)	<0.0001
LLP and PP	41/4260 (0.96 %)	6.63 (4.59–9.38)	<0.0001
Placenta accreta	28/412 (6.80 %)	47.8 (30.5–72.4)	<0.0001
Uterine rupture	9/182 (4.945 %)	31.0 (13.7–61.3)	<0.0001
Eclampsia	6/515 (1.17 %)	6.90 (2.49–15.4)	0.0003
Atonic bleeding	85/12,011 (0.71 %)	6.09 (4.57–8.07)	<0.0001
Uterine inversion	0/11 (0.00 %)	24.8 (0.193–191)^b^	0.1353
Birth canal laceration	1/136 (0.74 %)	4.25 (0.106–24.3)	0.2120

Critical bleeding was defined as a massive RBC transfusion (≥4000 cc) on the day of childbirth

*CI* confidence interval, *CS* Caesarean section, *HDP* hypertensive disorders of pregnancy, *HELLP* hemolysis, elevated liver enzymes, and low platelet count, *LLP* low-lying placenta, *OR* odds ratio, *PP* placenta previa

^a^Unadjusted OR

^b^Firth’s logistic regression analysis was performed for these variables with complete separation

### Multiple logistic regression analysis for critical bleeding

Based on the above findings, a multiple binary logistic regression analysis for the occurrence of critical bleeding was performed among the 354,299 women, simultaneously entering the following 17 explanatory variables into the model to determine the non-obstetric risks of critical bleeding: age; BMI; type of child delivery; HDP; HELLP syndrome; amniotic embolism; LLP/PP; placenta accreta; uterine rupture; atonic bleeding; uterine inversion; oral intake of prednisolone, anti-coagulants, or anti-platelet; hypertension; diabetes mellitus; iron deficiency anemia; and hypoproteinemia (Table 4). Regarding the demographic data, a higher age was significantly associated with critical bleeding (unit aOR per 1 year, 1.07; 95% CI, 1.05–1.09). Regarding the non-obstetric conditions, the use of oral prednisolone (aOR, 2.51; 95% CI, 1.44–4.40), oral anti-coagulants (aOR, 10.2; 95% CI, 5.44–18.9), and oral anti-platelets (aOR, 2.88; 95% CI, 1.29–6.40) and prenatal history of hypertension (aOR, 2.54; 95% CI, 1.46–4.40) and hypoproteinemia (aOR, 5.84; 95% CI, 1.72–19.9) had significant ORs >1.0. For reference, among the 4721 women with HDP, 70 (1.5%) had prenatal history of hypertension. The prevalence of HDP among those with prenatal history of hypertension (*n* = 70/4034; 1.7%) was slightly higher than that among those without prenatal history of hypertension (*n* = 4651/350,265; 1.3%; *p* = 0.0297, chi-square test).

**Table 4:** Multiple logistic regression analysis using obstetric and non-obstetric conditions for the development of critical bleeding

Characteristics	Critical bleeding, *n*^a^	Unadjusted results		Adjusted results		VIF
		OR (95% CI)	*P*-value	OR (95% CI)^b^	*P*-value	
Age (per 1 yr)	–	1.080 (1.061–1.099)	<0.0001	1.066 (1.047–1.086)	<0.0001	1.021
BMI (per 1.0)	–	0.958 (0.936–0.980)	0.0003	0.978 (0.954–1.002)	0.0739	1.032
Vaginal delivery (reference)	163/79,731	1.000	–	1.000	–	–
Elective CS	106/151,403	0.342 (0.265–0.440)	<0.0001	0.399 (0.300–0.530)	<0.0001	1.197
Emergent CS	215/123,165	0.854 (0.693–1.05)	0.1412	1.06 (0.831–1.35)	0.6524	1.157
HDP	13/4721	2.05 (1.08–3.54)	0.0164	2.04 (1.16–3.60)	0.0135	1.006
HELLP	17/1577	8.22 (4.74–13.3)	<0.0001	7.29 (4.37–12.2)	<0.0001	1.008
Amniotic embolism	31/79	498 (304–836)	<0.0001	252 (148–428)	<0.0001	1.001
LLP and PP	97/15,477	5.52 (4.37–6.91)	<0.0001	5.94 (4.59–7.69)	<0.0001	1.042
Placenta accreta	71/3175	19.4 (14.8–25.1)	<0.0001	7.34 (5.47–9.84)	<0.0001	1.022
Uterine rupture	17/230	60.4 (34.3–100)	<0.0001	55.4 (31.2–98.4)	<0.0001	1.001
Atonic bleeding	262/40,725	9.14 (7.61–11.0)	<0.0001	6.35 (5.22–7.72)	<0.0001	1.031
Uterine inversion	9/132	54.5 (24.2–107)	<0.0001	10.5 (4.91–22.4)	<0.0001	1.004
Oral prednisolone	17/3166	4.05 (2.34–6.57)	<0.0001	2.51 (1.44–4.40)	0.0012	1.011
Oral anti-coagulants	13/855	11.6 (6.09–20.1)	<0.0001	10.2 (5.44–18.9)	<0.0001	1.005
Oral anti-platelets	8/1047	5.71 (2.44–11.4)	0.0001	2.88 (1.29–6.40)	0.0096	1.013
Prenatal hypertension	16/4034	2.98 (1.69–4.89)	<0.0001	2.54 (1.46–4.40)	0.0009	1.011
Diabetes mellitus	4/4489	0.649 (0.176–1.67)	0.5394	0.657 (0.237–1.82)	0.4200	1.017
Iron deficiency anemia	75/56,321	0.970 (0.748–1.24)	0.8579	0.709 (0.545–0.922)	0.0104	1.028
Hypoproteinemia	4/128	23.8 (6.35–62.8)	<0.0001	5.84 (1.72–19.9)	0.0048	1.001

Binary logistic regression analysis was performed for the overall cohort (354,299 women) who had childbirth during the study period, using critical bleeding requiring ≥4000 cc of RBC transfusion as the dependent variable

*CI* confidence interval, *CS* Caesarean section, *HDP* hypertensive disorders of pregnancy, *HELLP* hemolysis, elevated liver enzymes, and low platelet count, *LLP* low-lying placenta, *OR* odds ratio, *PP* placenta previa, *VIF* variance inflation factor

^a^The shown numbers represent the numbers of cases with critical obstetric bleeding requiring an RBC transfusion of ≥4000 cc among the overall cohort with each condition

^b^Adjusted ORs were calculated with multivariable binary logistic regression analyses by simultaneously using all 18 explanatory variables (2 demographic, 9 obstetric, and 7 prenatal non-obstetric variables)

As there was a possibility that the multivariable analysis could have been influenced by the types of delivery, an additional multiple logistic regression analysis was performed among the 79,731 women who had vaginal childbirth (Table 5). In this analysis, prenatal hypertension showed a significant aOR (>1.0) for critical obstetric bleeding (aOR, 6.29; 95% CI, 2.27–17.5).

**Table 5:** Multiple logistic regression analysis for the development of critical bleeding among the 79,731 women who had vaginal childbirth

Characteristics	Critical bleeding, *n*^a^	Unadjusted results		Adjusted results		VIF
		OR (95% CI)	*P*-value	OR (95% CI)^b^	*P*-value	
Age (per 1 yr)	–	1.128 (1.093–1.163)	<0.0001	1.110 (1.073–1.148)	<0.0001	1.008
BMI (per 1.0)	–	0.978 (0.936–1.021)	0.3059	0.977 (0.933–1.022)	0.3100	1.017
Vacuum extraction	52/47,323	0.320 (0.226–0.449)	<0.0001	0.421 (0.291–0.608)	<0.0001	1.041
Episiotomy	14/12,358	0.512 (0.273–0.886)	0.0197	0.773 (0.424–1.409)	0.4002	1.017
HDP	1/914	0.532 (0.013–3.01)	>0.99	0.619 (0.085–4.505)	0.6358	1.005
HELLP	2/72	14.1 (1.66–53.8)	0.0097	18.9 (4.41–81.4)	<0.0001	1.001
Amniotic embolism	14/30	460 (207–1090)	<0.0001	279 (117–666)	<0.0001	1.001
LLP and PP	4/261	7.76 (2.07–20.5)	0.0021	2.19 (0.620–7.72)	0.2236	1.005
Placenta accreta	33/2126	9.40 (6.19–13.9)	<0.0001	3.95 (2.52–6.21)	<0.0001	1.022
Uterine rupture	8/19	370 (129–1034)	<0.0001	301 (97.0–934)	<0.0001	1.001
Atonic bleeding	112/14,630	9.84 (7.00–14.0)	<0.0001	6.40 (4.42–9.26)	<0.0001	1.051
Uterine inversion	9/109	46.4 (20.3–93.9)	<0.0001	16.8 (7.65–36.8)	<0.0001	1.006
Oral prednisolone	6/497	6.15 (2.21–13.8)	0.0006	2.46 (0.842–7.17)	0.0999	1.012
Oral anti-coagulants	2/110	9.14 (1.08–34.4)	0.0216	4.28 (0.750–24.5)	0.1019	1.006
Oral anti-platelets	3/92	16.7 (3.35–51.3)	0.0009	9.56 (2.19–41.7)	0.0027	1.015
Prenatal hypertension	5/348	7.31 (2.33–17.6)	0.0008	6.29 (2.27–17.5)	0.0004	1.005
Diabetes mellitus	1/589	0.829 (0.021–4.70)	>0.99	0.815 (0.111–5.99)	0.8406	1.008
Iron deficiency anemia	18/7322	1.23 (0.708–2.01)	0.492	0.644 (0.368–1.14)	0.1231	1.016

In this regression model among women who had vaginal childbirth, the delivery type variable was substituted with the performance of vacuum extraction and episiotomy. Hypoproteinemia was excluded from the model as no women who had vaginal childbirth had both conditions of hypoproteinemia and massive RBC transfusion

^a^The shown numbers represent the numbers of cases with critical obstetric bleeding requiring an RBC transfusion of ≥4000 cc among the overall cohort with each condition

^b^Adjusted ORs were calculated with multivariable binary logistic regression analyses by simultaneously using all 18 explanatory variables (2 demographic, 10 obstetric, and 6 prenatal non-obstetric variables)

Furthermore, as the use of BMI during hospitalization as a confounding factor may not be suitable in these two logistic regression models, the analyses were performed after removing BMI from the explanatory variables. The statistical significance of the aOR for prenatal hypertension did not change in either of the regression models among the overall population (aOR, 2.55; 95% CI, 1.50–4.35; *p* = 0.0006) or among those who had vaginal childbirth (aOR, 5.42; 95% CI, 1.97–14.9; *p* = 0.0010).

### Impact of prenatal hypertension on each obstetric complication

Based on the finding that a prenatal history of hypertension was significantly associated with the occurrence of critical bleeding in obstetrics in both univariate and multivariate analyses, the effect of prenatal hypertension on the presence of each obstetric complication was further evaluated by calculating the unadjusted and adjusted ORs of prenatal hypertension for subsequently developing each obstetrical complication during childbirth (Table 6). A prenatal history of hypertension had significant aOR (>1.0) for DIC (aOR, 1.92; 95% CI, 1.53–2.40), HELLP syndrome (aOR, 3.33; 95% CI, 2.67–4.15), and eclampsia (aOR, 6.14; 95% CI, 4.62–8.14). Meanwhile, non-significant aORs were observed for HDP (aOR, 1.07; 95% CI, 0.85–1.36), placental abruption (aOR, 1.19; 95% CI, 0.95–1.49), amniotic embolism (aOR, 2.90; 95% CI, 0.91–9.27), LLP/PP (aOR, 0.50; 95% CI, 0.41–0.61), placenta accreta (aOR, 1.02; 95% CI, 0.70–1.50), uterine rupture (aOR, 0.25; 95% CI, 0.04–1.81), atonic bleeding (aOR, 1.00; 95% CI, 0.91–1.11), uterine inversion (aOR, 1.21; 95% CI, 0.17–8.68), and birth canal laceration (aOR, 0.79; 95% CI, 0.55–1.14).

**Table 6:** Multiple logistic regression analyses using prenatal history of hypertension for occurrence of each obstetric complication

Obstetrical complications	Prenatal HTN, *n* (%)^a^	Unadjusted results		Adjusted results	
		OR of HTN (95% CI)	*P*-value	OR of HTN (95% CI)^b^	*P*-value
Total	4034/354,299 (1.1%)	NA	NA	NA	NA
DIC	80/3099 (2.6%)	2.33 (1.84–2.91)	<0.0001	1.92 (1.53–2.40)	<0.0001
HDP	70/4721 (1.5%)	1.31 (1.02–1.67)	0.0297	1.074 (0.846–1.36)	0.5574
HELLP	87/1577 (5.5%)	5.16 (4.10–6.42)	<0.0001	3.33 (2.67–4.15)	<0.0001
Placental abruption	78/4116 (1.9%)	1.69 (1.33–2.12)	<0.0001	1.19 (0.946–1.49)	0.1376
Amniotic embolism	3/79 (3.8%)	3.43 (0.692–10.4)	0.0617	2.90 (0.905–9.27)	0.0731
LLP and PP	102/15,477 (0.66%)	0.565 (0.459–0.688)	<0.0001	0.501 (0.411–0.610)	<0.0001
Placenta accreta	27/3175 (0.85%)	0.743 (0.488–1.08)	0.1461	1.02 (0.696–1.50)	0.9175
Uterine rupture	1/230 (0.43%)	0.379 (0.010–2.13)	0.5295	0.253 (0.035–1.81)	0.1706
Eclampsia	54/738 (7.3%)	6.93 (5.15–9.17)	<0.0001	6.14 (4.62–8.14)	<0.0001
Atonic bleeding	423/40,725 (1.0%)	0.901 (0.812–0.997)	0.0460	1.004 (0.907–1.11)	0.9375
Uterine inversion	1/132 (0.76%)	0.663 (0.017–3.76)	>0.99	1.21 (0.168–8.68)	0.8514
Birth canal laceration	32/8937 (0.36%)	0.307 (0.209–0.434)	<0.0001	0.793 (0.552–1.14)	0.2093

The unadjusted and adjusted ORs of the history of prenatal hypertension for subsequently developing each of the listed obstetric complications during the childbirth were calculated among the overall 354,299 women

*CI* confidence interval, *DIC* disseminated intravascular coagulation, *HDP* hypertensive disorders of pregnancy, *HELLP* hemolysis, elevated liver enzymes, and low platelet count, *HTN* hypertension, *LLP* low-lying placenta, *OR* odds ratio, *PP* placenta previa

^a^Percentages were calculated as the number of women with a prenatal history of hypertension among those with each obstetric complication

^b^Adjusted OR of prenatal hypertension for later developing each obstetric complication was calculated by performing a multiple logistic regression analysis using (1) age, (2) type of delivery, and (3) prenatal history of HTN as explanatory variables. The presence of each obstetric complication was used as the dependent variable

**Fig. 1:**
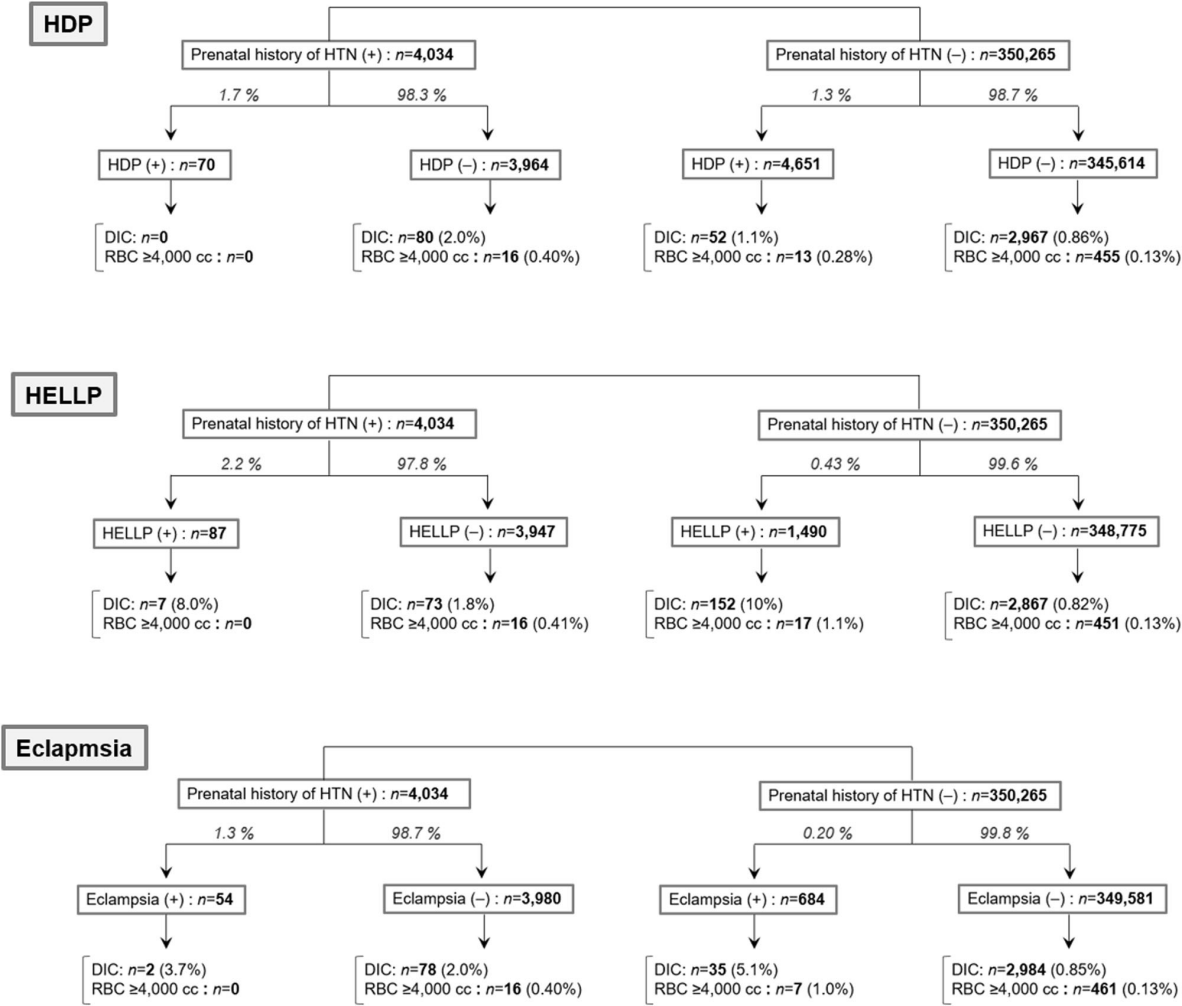
Development rate of DIC and critical bleeding in HELLP and eclampsia groups with respect to prenatal history of hypertension. The developmental rates of DIC and critical obstetric bleeding in the HDP, HELLP, and eclampsia groups are shown with respect to the presence of a prenatal history of hypertension. The prevalence of HDP increased only slightly in the presence of prenatal hypertension. The prevalence of HELLP syndrome and eclampsia increased by more than five times in the presence of prenatal hypertension. Meanwhile, the rates of subsequent development of DIC and critical bleeding in the HELLP and eclampsia groups did not differ in the presence of prenatal hypertension, suggesting that prenatal hypertension may predispose critical obstetric bleeding via these obstetric conditions. DIC disseminated intravascular coagulation, HDP hypertensive disorders of pregnancy, HELLP hemolysis, elevated liver enzymes, and low platelet count, RBC red blood cell

Based on these findings, the occurrence rates of HDP, HELLP, and eclampsia among the overall cohort were investigated after dividing the cohort based on the presence of a prenatal history of hypertension. In all subgroups, the subsequent occurrence rates of DIC and critical bleeding requiring an RBC transfusion of ≥4000 cc were further investigated (Fig. 1). The rate of HDP development was only slightly higher in patients with prenatal history of hypertension. The rates of developing HELLP and eclampsia were more than five times higher among patients with prenatal history of hypertension. Meanwhile, the subsequent development rates of DIC and critical bleeding in the HELLP and eclampsia groups did not increase in the presence of a prenatal history of hypertension.

## Discussion

In this study, we evaluated the impact of maternal age and several common non-obstetric conditions on the development of critical bleeding in obstetrics requiring a massive RBC transfusion (≥4000 cc). Both univariate and multivariate analyses indicated that the significant risks underlying critical obstetric bleeding included higher maternal age; medication with oral steroids, anticoagulants, and antiplatelets; and a prenatal history of hypertension and hypoproteinemia. These findings were confirmed even after adjusting for the type of delivery and the coexistence of obstetric complications predisposing to critical bleeding. Prenatal history of hypertension was not associated with subsequent atonic bleeding (OR, 1.00; 95% CI, 0.91–1.11). Meanwhile, prenatal hypertension was associated with HELLP syndrome (OR, 3.3; 95% CI, 2.7–4.2) and eclampsia (OR, 6.1; 95% CI, 4.6–8.1), the former was identified as a significant risk of critical obstetric bleeding among those who had either of the three types of delivery and the latter was identified as a risk among those who had an emergent CS. Collectively, these findings suggest possible common conditions underlying prenatal hypertension, HELLP syndrome, eclampsia, and critical obstetric bleeding.

Although this study revealed that prenatal history of hypertension is associated with the development of HELLP syndrome and eclampsia, the subsequent development rates of DIC and critical bleeding requiring a massive RBC transfusion (≥4000 cc) in HELLP and eclampsia groups did not differ in the presence of prenatal hypertension. The present study found that the presence of prenatal hypertension is a significant predictor of HELLP, eclampsia, and the subsequent development of DIC and critical obstetric bleeding. However, the above-mentioned findings imply that prenatal hypertension is not the direct cause of obstetric DIC and critical obstetric bleeding. DIC and critical obstetric bleeding are caused by underlying multiple-organ vascular damage and dysfunction, not simply by high blood pressure itself. Further studies are needed to elucidate the mechanisms connecting the three common overlapping conditions (i.e., chronic hypertension, HELLP syndrome, and eclampsia) with critical obstetric bleeding.

The clinical and laboratory manifestations of HELLP syndrome and preeclampsia-eclampsia differ; however, these two conditions are considered to fall within obstetric hypertensive disorders [23]. HELLP syndrome, preeclampsia-eclampsia, and HDP are all considered to result from vascular dysfunction, such as widespread vasospasm caused by multi-organ endothelial vascular damage in different organs or systems, during pregnancy [24–26]. Certainly, in the cohort of the present study, the overlapping of HELLP and eclampsia was likely to be seen in both populations with HELLP (*n* = 48/1577; 3.0%) and eclampsia (*n* = 48/738; 6.5%). The findings of the present study are compatible with the disease concept based on vascular dysfunctions incorporating HELLP syndrome, eclampsia, and hypertension. This vascular triad is a life-threatening condition that predisposes patients to critical obstetric bleeding. Additionally, the present study suggests that this vascular triad is not associated with the occurrence of atonic bleeding, which is another common cause of critical obstetric bleeding [27–29]. Different mechanisms underlying obstetrical critical bleeding are implied; (1) conditions with vasospasm resulting in the development of aforementioned vascular triad and (2) conditions with other causes bringing atonic postpartum hemorrhage including functional and traumatic causes.

Another notable finding of this study was that the expected maternal mortality rate surpassed 1.0% among those who received a massive RBC transfusion (≥4000 cc), which could rationalize the use this amount as the cutoff to determine critical bleeding in obstetrics. The majority of the women who received an RBC transfusion of <20,000 cc survived the critical bleeding, whereas the majority of those who received an RBC transfusion of ≥20,000 cc did not survive. These findings imply that an RBC transfusion of 4000 cc would be the optimal cutoff for deciding a critical level, and that 20,000 cc would be an indicator of a lethal level. Since it is difficult to measure the exact amount of bleeding during childbirth, paying careful attention to maternal vital signs and referring to derivative indices, such as the SI, would benefit obstetricians predict life-threatening bleeding early. Notably, none of the five women (including four women who did not survive) who required an RBC transfusion of ≥20,000 cc underwent total hysterectomy, whereas all of them were administered platelets and FFP. This finding implies that an early decision to perform hysterectomy may contribute to saving maternal life once the RBC transfusion surpasses a certain amount, such as 10,000 or 15,000 cc, before the maternal condition becomes too dangerous to undergo surgery. Interventional radiology (IVR), which is minimally invasive, free of the risk of general anesthesia, and fertility-preserving, is another available therapeutic option for controlling massive obstetric hemorrhage [30, 31]. Currently, few hospitals implement IVR worldwide, and there is an urgent need to establish systems to ensure that all mothers have access to IVR as a first-line therapeutic option for intractable critical obstetric hemorrhage [31].

The present study demonstrated that a prenatal history of hypoproteinemia/hypoalbuminemia significantly increased the risk of critical obstetric hemorrhage, with a calculated crude OR of 23.8. In previous studies that enrolled patients with non-valvular atrial fibrillation who were taking warfarin, hypoalbuminemia was confirmed to be a significant risk factor for major bleeding events, especially in the younger population aged <75 years [32, 33]. The authors of these previous studies proposed several theories to explain this association, such as a predisposition to bleeding or recent subclinical hemorrhage. Another more recent study evaluating patients admitted to the intensive coronary care unit also confirmed an increase in major bleeding events during hospitalization among patients with low albumin level and described that the pathophysiology of hypoalbuminemia is diverse and multifactorial [34]. Hypoalbuminemia is often a secondary consequence of other diseases, and some of these conditions can be treated with proper medications [35–37]. Further studies are needed to establish an appropriate therapeutic strategy to prevent critical obstetric hemorrhage in pregnant women with prenatal hypoalbuminemia.

This study has several limitations. First, the evaluated population did not exactly represent the overall number of pregnant women in Japan during the study period because the administrative DPC database did not cover the majority of women who had vaginal childbirth. All women who had an elective or emergent CS at the investigated DPC hospitals were available, but those who had delivered their children without any complications, interventions, or medications did not appear in the DPC payment system; therefore, the database did not include data of these women. Since all women who developed critical obstetric bleeding are considered to be included in the DPC database, the actual estimated ORs and the 95% CI among women who had vaginal childbirth would be lower than those calculated in the present study. Second, the control level of blood pressure before and during pregnancy was not available for each woman. Further studies are needed to determine the optimal home blood pressure level during pregnancy to efficiently suppress the risk of critical obstetric bleeding. Finally, the racial or ethnic identity of each woman in the database was not available; therefore, it is unclear whether the findings of this study can be generalized to other countries and regions with different ethnic compositions.

In conclusion, higher maternal age; prenatal history of hypertension and hypoproteinemia; and conditions requiring oral prednisolone, oral anticoagulants, and oral antiplatelets were identified as significant risk factors underlying critical obstetric bleeding, together with other obstetric complications. Prenatal hypertension may predispose patients to HELLP syndrome, eclampsia, and subsequent development of critical bleeding, but it may not predispose the patients to atonic bleeding. Mothers with these obstetric and non-obstetric risks of critical bleeding may benefit from additional caution to avoid overlooking persistent bleeding, and to ensure swift interventions with efficient transfusion therapy, hemostatic procedures, and damage control surgeries.

## Data Availability

Individual-level data were not available because of the agreement with the contributing facilities and approval of the institutional review boards. Anonymized data supporting the findings of this study and the detailed study protocol are available from the corresponding author upon reasonable request.
